# Treatment of Pulpless Teeth Having No Fistulous Opening

**Published:** 1895-06

**Authors:** C. N. Johnson

**Affiliations:** Chicago.


ARTICLE VII.

### TREATMENT OF PULPLESS TEETH HAVING NO FISTULOUS OPENING.

DR. C. N. JOHNSON, CHICAGO.

In the treatment of teeth with pulps dead but with no fistulous opening, where there is any doubt as to whether a blind abscess exists it should invariably be treated as if there were one. The most expert operator cannot always determine in advance whether he has a case of blind abscess or simply a putrescent pulp-canal with no complications beyond the apex, and it is only common justice to the patient to proceed in the most cautious manner. In fact, these require the most careful management of any form of pulpless teeth.

There are two principal conditions calling for treatment under this head: Where the pulps have died under fillings or in sound teeth, and where decay has exposed the pulp and caused its death. In the former the operator must drill in the tooth to gain access to the chamber and canals, and in this connection one precaution seems always necessary. Formerly these frequently resulted in much trouble to the patient and chagrin to the operator, through the fact that teeth which, previous to the interference on the part of the dentist, had been perfectly comfortable, immediately took on inflammatory action and often ran to a distressingly bad abscess. The precaution necessary to avoid such a result relates to thorough antisepsis from the very beginning of the operation. The rubber-dam should be applied before any drilling is attempted, and an antiseptic placed near at hand for immediate use the moment the pulp-chamber is penetrated. This antiseptic should be non-irritating and -non-effervescing. It should be a non irritant, because irritation either medicinal or mechanical is dangerous, and it, above all other things should not be an effervescing agent, because the very act of effervesence is too likely to force septic matter lying in the canal through in the apical space. The least disturbance we set up in the apical third of the canal at this first sitting the greater our chances of avoiding trouble, and I am much opposed to the practice advocated by various writers of using hydrogen peroxid, pyrozone, or similar agents thus early in the treatment of pulpless teeth having no fistulous openings.

As soon as the chamber is reached by the drill, it should at once be flooded with the antiseptic, care being taken not to cause any pressure on the contents of the canals. The slightest particle of septic matter forced through the apical foramen at this stage of the operation may cause serious trouble. When the antiseptic has had time to mix somewhat with the contents of the chamber, a bit of absorbent cotton or bibulous paper may be lightly applied to the opening to absorb enough of the antiseptic to admit of drilling to enlarge the opening. The absorbent will usually bring away some of the putrescent contents of the chamber in addition to the antiseptic, and when the opening is sufficiently enlarged the chamber may repeatedly be flooded, and the contents absorbed in this way till the greater part of putrescent matter has been removed from the chamber and the larger portion of the canals without causing disturbance at the apical foramen, When this process has been carried to the limit of safety, the cavity is flooded with absolute alcohol for its dehydrating effect, and this evaporated to dryness. This step in the operation seems to the writer to be of the utmost importance. It must be manifest that the dryer we get the canals the less chance there is for micro-organic life to exist, and that when an antiseptic is applied the greater will be the amount taken up by the tubuli. Care should be exercised, however, that the crown of the tooth be not heated too hot in the process of drying the root, and that it be not kept dry too long, for fear of injury to the tooth-structure through checking. It is with this possible danger that I have nearly abandoned the use of hot air in these cases and substituted the alcohol spray. It seems to extract moisture from the canals without heating the tooth perceptibly. This danger to the crown of the tooth may also be considered an argument in favor of the use of the root-canal drier whenever it is found practicable, and wherever it is felt that there is no danger of causing apical irritation by its use. When dryness of the canals is obtained, they should once more be flooded with an antiseptic, this time to remain sealed in the cavity till the next sitting. The canals are left filled with the medicament, but no cotton is forced in them at this time. A small pledget saturated with the antiseptic may be placed loosely in the chamber, and the opening closed with gutta-percha.

If the operation is carried through thoroughly and carefully, and if the putrescence seems to have been overcome by the treatment, dismissed for one week, with instructions to return at once if trouble ensues. Where any doubt is felt as to the degree of control obtained, the patient should be seen in twenty-four or forty-eight hours, and the dressing changed. At the end of a week, if no symptoms of irritation have been felt, and if the canals are sweet and clean on the removal of the dressing, the roots may be filled. Before this is done, however, the canals should first be washed out with alccohol, dried, flooded once more with an antiseptic, wiped out, flooded with alcohol, and dried. They are then ready for the filling.

Where the pulp has died as a result of exposure from decay, and the canals have for long been subject to septic influence, the same general treatment may be followed, with the one slight variation that in extensive decay, with much infected and softened dentine remaining, the softened portion may be burred out or removed with excavators, and washed away with a syringe before the rubber-dam is applied. In this way much filth may conveniently be gotten rid of by way of the spittoon without the operator coming in too close contact with it.

In treating where there are no fistulous openings, if the dressing after the first treatment shows indications that there is a blind abscess through a weeping of pus in the canal, the treatment should be continued till all such weeping stops and the abscess heals. In chronic, persistent blind abscess, where large quantities of pus flow in the canal and flood the entire cavity on removal of the dressing, the treatment is to remove all of the pus possible by absorbing with cotton or bibulous paper, and by "coaxing" with a broach. When pus is flowing in such profusion it is an indication that the apical foramen is large, and there is less danger of causing irritation by the use of the broach in this region than in ordinary canals without a flow of pus. It is therefore advisable to manipulate with the broach quite freely in the attempt to empty the abscess of all pus, and sometimes desirable to carry this manipulation to the point where pus stops and blood begins to flow. When the operator succeeds in getting a good flow of blood, he may then wipe the canal with a broach wrapped with cotton and dipped in some non-irritating antiseptic, and when the canal is sufficiently free from blood, it may be packed to the apex with cotton saturated with the antiseptic. This should be allowed to remain a week undisturbed if it causes no uneasiness. Nature should be given time to repair the injury, and nature will very often do much for us if we give her the opportunity. This is where much harm is often done by over-treatment.

If a tooth in this condition will remain tightly packed for a week without trouble, and if the canal is dry and the dressing sweet on removal, the root may be filled.—*Cosmos.*

---

### Communicated.

